# Development of Drought-Tolerant Transgenic Wheat: Achievements and Limitations

**DOI:** 10.3390/ijms20133350

**Published:** 2019-07-08

**Authors:** Shahbaz Khan, Sumera Anwar, Shaobo Yu, Min Sun, Zhenping Yang, Zhi-qiang Gao

**Affiliations:** 1 College of Agriculture, Shanxi Agricultural University, Taigu 030801, China; 2 State Key Laboratory of Rice Biology, China National Rice Research Institute, Hangzhou 310006, China

**Keywords:** genetic modification, *Triticum aestivum* L., water stress, transcription factors

## Abstract

Crop yield improvement is necessary to keep pace with increasing demand for food. Due to climatic variability, the incidence of drought stress at crop growth stages is becoming a major hindering factor to yield improvement. New techniques are required to increase drought tolerance along with improved yield. Genetic modification for increasing drought tolerance is highly desirable, and genetic engineering for drought tolerance requires the expression of certain stress-related genes. Genes have been identified which confer drought tolerance and improve plant growth and survival in transgenic wheat. However, less research has been conducted for the development of transgenic wheat as compared to rice, maize, and other staple food. Furthermore, enhanced tolerance to drought without any yield penalty is a major task of genetic engineering. In this review, we have focused on the progress in the development of transgenic wheat cultivars for improving drought tolerance and discussed the physiological mechanisms and testing of their tolerance in response to inserted genes under control or field conditions.

## 1. Introduction

Bread wheat (*Triticum aestivum* L.) is one of the most important cereal and staple food crops worldwide, mainly grown in semiarid and arid regions of the world where water scarcity is causing severe yield losses [1]. The constantly increasing population and land degradation have increased the desire for per acre yield increase [2]. It is estimated that the global wheat production should increase approximately 60% from the current 3.3 t ha^−1^ to 5 t ha^−1^ by 2050 to feed a population of 9 billion [3,4]. However, wheat production is severely affecting and reducing by 29% due to various abiotic stresses, especially drought stress [5,6].

Therefore, improving the tolerance of wheat to drought stress through adaptive strategies is important to ensure food security. To achieve this goal without increasing the area of cultivated land, which is simply not available, emphasis must be concentrated on key traits related to plant productivity and adaptation to environmental challenges. Genetic improvement and developing drought-tolerant wheat cultivars is critically important and a main aim for wheat breeders [7]. Different techniques such as marker-assisted breeding, quantitative trait locus mapping, and introgression from the wild gene pool are being employed to improve drought tolerance [8,9,10]. Various genes of interest could be inserted by genetic transformation. In contrast to conventional breeding, transgenic technique seems to be a more attractive approach which allows the direct induction of a single or group of desired genes [11].

Drought tolerance is a complex quantitative polygenic trait controlled by a large number of genes and thus, it is difficult to understand the molecular and physiological mechanisms [1,12]. Until now, genetically-modified *Glycine max* (soybean) and *Zea mays* (maize) for drought tolerance have been developed and approved. However, despite continuous research, less progress has been made for the development of transgenic wheat as compared to other staple crops like rice and maize which might be ascribed to the complex genetic characteristics of wheat [13].

Although transgenic plants have a potential to transform agriculture, limited progress has been achieved, particularly for wheat where no drought-tolerant transgenic wheat has been approved for commercialization [13]. The first fertile herbicide-resistant transgenic wheat plant was obtained by microprojectile bombardment of regenerable embryonic callus [14]. The first approved transgenic wheat for glyphosate herbicide tolerance was developed by Monsanto. It is an important step forward in an area where progress is urgently needed, though it is too early to claim that transgenic wheat will form the backbone of a second Green Revolution [15].

The task of the successful generation of transgenic plants is not only limited to the success in the transformation process but also to properly incorporate the stress tolerance. The proper understanding of physiological effects of the inserted gene and evaluation of transgenic plants under stress are major challenges. After controlling and inserting ‘single-action’’ genes, the regulatory mechanism of transcription factors has emerged as a new tool for controlling the expression of many stress-responsive genes. González et al. [16] reported that transgenic wheat transformed with mutated transcription factor (*HaHB4*) from *Helianthus annuus* (sunflower), which belongs to homeodomain-leucine zipper family (HD-Zip I), and had increased water use efficiency and yield. Transgenic wheat carrying a *GmDREB1* gene from soybean, under a ubiquitin promoter, showed drought tolerance [17]. One main interest is to improve WUE so plants could grow well under water stress. Water use efficiency could be improved by genetically engineering in two ways. One way is inserting genes for compatible osmolytes such as sugar and amino acids [18]. The other strategy is constitutive over-expression of the late embryogenic abundant proteins which provide dehydration tolerance.

This review focuses mainly on the recent studies of genes involved in physiological or biochemical processes which have been used successfully in the genetic engineering of wheat for improvement of drought tolerance (summarized in Table 1). Furthermore, we will discuss recent successes and limitations in the application of genetic manipulation to increase drought tolerance in wheat.

## 2. Drought-Induced Gene Expression/Single Action Gene

Numerous genes have been isolated from various plants and inserted in transgenic wheat to induce stress resistance. These genes could be classified into two groups. The first group is genes involved in cellular protection including osmoprotectants, membrane stabilization, detoxification, and transport proteins. The genes in the second group are the transcription factors and signaling molecules [19].

## 3. Osmoprotectants, Metabolites and Protective Genes

These osmoprotectants are the favorite targets for genetic engineering and many crops are genetically engineered using osmoprotectants, like glycine betaine, mannitol, and trehalose to increase tolerance by protecting important macromolecules. The higher accumulation of these compounds by transferring genes in transgenic wheat plays a critical role in improving drought tolerance [20]. Higher tolerance is by osmotic adjustment and chaperone-like activity in stabilizing membrane and protein and detoxification by scavenging reactive oxygen species [21].

### 3.1. Proline

Proline is a well-known proteogenic amino acid which acts as a compatible osmoprotectant and accumulates under osmotic stress to protect cellular structure and function [22,23]. Proline under stress works as an antioxidant and scavenges reactive oxygen species, protects denaturation of macromolecules, and regulates cytosolic activity [24]. The accumulation of proline contents in plants is correlated with the tolerance to drought stress; therefore, the overexpression of proline in transgenic plants increased stress tolerance in wheat [25], potato (*Solanum tuberosum* L.) [23] and tobacco (*Nicotiana tabacum*) [26]. In plants, proline is synthesized from glutamate through the glutamate pathway. The reduction of glutamate to its semialdehyde is catalyzed by P5CS (∆^1^-pyrroline-5-carboxylate synthase) enzyme, which then reduces to proline. P5CS is a rate-limiting enzyme for the synthesis of proline by feedback inhibition of P5CS [27].

Vendruscolo et al. [19] used the biolistics transformation method to transform cDNA of the P5CS gene of *Vigna aconitifolia* under stress-inducible AIPC promotor. Transgenic wheat plants were exposed to 14 days of water withholding at the booting stage. Transgenic plants showed 50% intact cellular membrane as compared to wild type with 13% intact membrane. The enhanced tolerance to water deficit was mainly due to improved antioxidants also manifested with less MDA contents, rather than the enhanced osmotic adjustment. Pavei et al. [28] evaluated seven transgenic lines in T_2_ generation containing the p5cs gene, and transgenic plants produced 1.85 times more proline after 8 days of water stress content than non-transgenic showing the overexpression of the p5cs gene. However, MDA contents were not decreased in transgenic plants which indicated that despite higher proline accumulation, cell membrane protection against oxidative stress was not increased.

### 3.2. Glycine Betaine

Glycine betaine is a quaternary ammonium compound and is known to have a protective role against drought stress by maintaining osmotic balance and protecting quaternary structures of proteins [29]. Various genes (BADH, COD, CDH, and *betaA*) involved in the synthesis of glycine betaine, have been inserted into transgenic plants [30,31,32]. The gene encoding betaine aldehyde dehydrogenase enzyme (BADH) synthesizes glycine betaine by catalyzing betaine aldehyde into glycine betaine. However, transgenic plants with glycine betaine synthesizing genes could accumulate lower levels of glycine betaine than natural accumulators, still enhancing the tolerance to various abiotic stresses [33,34]. Transgenic wheat lines overexpressing BADH showed enhanced tolerance to salt stress especially by protecting the thylakoid membrane [34].

The wheat plants naturally accumulate glycine betaine, but the in-vivo level is too low to maintain the osmotic balance under drought stress [29,33]. Transgenic wheat transformed and cloned containing cDNA of BADH from *Atriplex hortensis* showed higher betaine aldehyde dehydrogenase activity [35]. Wang et al. [36] generated T_6_ transgenic wheat line overexpressing the BADH gene cloned from *Atriplex hortensis* under maize ubiquitin promoter through the microprojectile bombardment method. Transgenic and wild type (Shi 4185) were exposed to osmotic stress at −1.88 MPa using 30% PEG and results indicated that transgenic plants overexpressing glycine betaine led to increased photosynthesis by improving water status and antioxidant activity. In the second study, T_6_ lines of transgenic wheat plants were exposed to drought (78–82% relative water content) by controlling irrigation [31]. Transgenic plants increased glycine betaine production and enhanced plant tolerance to drought by improving photosynthesis. In another study, transgenic wheat was produced by introducing *betA* gene-encoding choline dehydrogenase under maize ubiquitin promoter [32]. Transgenic plants under drought stress (12–14% relative water content) were less injured and showed higher root length and biomass which indicated that glycine betaine reduced the extent of the injury and worked as an important osmoprotectant in transgenic wheat under drought stress.

### 3.3. Mannitols

Mannitol is sugar-alcohol, which serves as a major carbon source and has a role in osmoregulation as a coenzyme regulator and scavenging of reactive oxygen species. Mannitol is widely distributed in various plant species [21]. Transgenic plants for *mtlD* gene from *Escherichia coli* in tobacco [37,38], rice (*Oryza indica*) [39], arabidopsis (*Arabidopsis thaliana*) [40] and peanut (*Arachis hypogaea* L.) [41] demonstrated improved photosynthesis and ROS scavenging activity and enhanced the tolerance to abiotic stresses [21,42]. Wheat plants naturally do not accumulate mannitol, although the higher expression of *mtlD* gene transformed from *Escherichia coli* caused the accumulation of mannitol in transgenic wheat [18]. Moderate accumulation of mannitol enhanced the tolerance to water stress, however, several abnormalities were also observed in transgenic plants due to short and sterile plants, twisted leaves and low sucrose contents [18]. Transgenic wheat plants with the accumulation of mannitol showed higher biomass, plant height, and flag leaf length as compared to non-accumulating [18].

### 3.4. Heat/Cold Shock Protein Chaperons/Molecular Chaperons

Bacterial cold shock proteins (CSP_S_) are important bacterial genes for bacterial acclimatization to low temperature and drought stress. CSP_S_ genes conferred resistance to drought stress and improved grain yield in maize under drought conditions [43]. Yu et al. [44] (2017) conducted a field experiment using transgenic wheat lines transformed with SeCspA and SeCspB genes from *Escherichia coli*. Transgenic wheat lines with *SeCspA* showed enhanced tolerance with lower water loss, less wilting and MDA content, and higher proline, chlorophyll content, 1000-grain weight, and grain yield compared to non-transgenic under drought stress. Transgenic lines with *SeCspB* showed no such improvement under field condition. Seven-day-old seedlings were exposed to severe water deficit and the recovery rate of *SeCspA* transgenic plant after re-watering was significantly higher than the non-transgenic plants [44].

### 3.5. Late Embryogenesis Abundant (LEA) Proteins

Late embryogenesis-abundant (LEA) proteins are hydrophilic and are induced by drought, salt, and ABA. LEA acts as a molecular chaperone protein by stabilizing the protein or membrane structure. HVA1 is an ABA-inducible LEA protein which is naturally accumulated in the aleurone layer of barley under seed desiccation [45]. Transgenic plants overexpressing *HVA1* gene in rice [46] *OsLEA3-1* in rice [47], and *HVA1* in spring wheat [48] enhanced abiotic stress tolerance.

Transgenic wheat over-expressing *HVA1* gene, which is a group-3 LEA protein, indicated the potential of the *HVA1* gene to enhance the tolerance to water deficit stress [48,49]. The barley *HVA1* gene transformation in transgenic wheat and its constitutive expression much improved the drought tolerance in terms of higher dry mass accumulation, WUE and higher cell integrity under moderate water deficit conditions in greenhouse tests [48]. After this, Bahieldin et al. [49] conducted nine field experiments to test the drought tolerance of *HVA1* transgenic wheat using T4 progeny of six transgenic lines and two new lines. Substantial variability was found for transgenic lines and coincided with the expression rate of *HVA1* transgene. Line 111/1 showed higher yield and biomass than the wild type, and in sixth season, the yield of new transgenic lines (765 and 1201) increased more than in line 111/1 and wild type. Higher relative water content was observed in lines with higher expression of the *HVA1* gene which shows the potential of the HVA1 gene to confer drought tolerance.

## 4. Transporters Genes

Transporters genes play an important role in restoring ionic homeostasis under stress. Ferritins are iron-storage proteins which sequester and release iron when needed. Ferritins are highly conserved in plants with two genes in hexaploid wheat on chromosomes 4 and 5 [50]. Previous studies showed that the over-expression of ferritin gene induced tolerance to various abiotic stresses like cold, heat and drought stresses [50,51]. Zang et al. [51] studied the response of transgenic wheat over-expressing TaFER-5B under various abiotic stresses. TaFER-5B from thermo-tolerant wheat ‘TAM107′ was transformed into cultivar ‘JM5265′ under maize ubiquitin promoter via the particle bombardment method. TaFER-5B transgenic wheat lines had higher total root length, whereas GR enzyme activity and H_2_O_2_ contents were lower under 10% PEG.

## 5. Carbon Metabolism

Transforming C_4_ enzymes genes is also a promising approach to increasing the yield and photosynthesis rate of C_3_ plants like wheat. Transgenic wheat plants over-expressing C_4_ enzymes, PEPC (phosphoenolpyruvate carboxylase) and PPDK (pyruvate orthophosphate dikinase), showed a significant positive effect on photosynthetic traits and yield [52]. Later, transformed wheat-expressing PEPC gene from maize was studied for drought tolerance [53]. Transgenic plants had higher yield, photosynthesis, soluble sugar, and proline contents and improved water use efficiency with more roots under drought stress. The increased photosynthesis in transgenic wheat was attributed to higher PEPC responsive proteins such as pyruvate, chlorophyll binding proteins, and phosphate dikinase enzymes.

## 6. Transcription Factors

Plants induce the expression of various transcription factors to cope with environmental stresses. Then the transcription factors up or down-regulate the expression of series of genes by binding with the enhancer or promoter region of the gene with DNA-binding domains [54,55,56]. Several transcription factors families like WRKY, DREB, MYB, NAC, and ERF are involved in the plants in response to drought stress by controlling the expression of stress-related genes [9,56,57,58]. Engineering transcription factor is an effective and practical approach to improve abiotic stress tolerance by genetic engineering. Previous studies indicated that transgenic plants overexpressing transcription factor-encoding genes could increase tolerance to abiotic stresses [59,60,61]. Transcription factors involved in drought tolerance could be utilized to develop drought-tolerant wheat cultivars [56].

### 6.1. DREB

Dehydration-responsive element binding (DREB) transcription factors have been reported to enhance drought tolerance in transgenic wheat [62,63]. In wheat, the *TaDREB1* is a DREB2-type transcription factor induced by drought and other abiotic stresses [64]. Likewise, a DREB-related gene, *TdDRF1*, has been isolated from durum wheat and is characterized to be involved in drought tolerance [65].

Wheat transgenic plants with the *DREB1A* gene were produced at CIMMYT and were tested in small pots under greenhouse [62]. *DREB1A* gene of *Arabidopsis thaliana* was transferred into bread wheat under the control of the stress-inducible rd29A promoter. Transgenic wheat plants expressing the *DREB1A* gene demonstrated substantial resistance to water stress as compared to wild type under greenhouse conditions, as manifested by delayed wilting and leaf bleaching after withholding water. On the other hand, the non-transgenic plants were dyed after 15 days of holding water [62]. The higher drought tolerance of the *AtDREB1A* transgenic plant is achieved by increasing the relative water content, chlorophyll, sugar, and proline contents as compared to non-transgenic plants [66].

Shiqing et al. [17] transformed the *GmDREB* gene of soybean into wheat by bombardment. Transgenic wheat plants using ubiquitin and rd29A promoters showed normal growth and higher drought resistance than the wild type. Furthermore, some transgenic seeds germinate at 18% PEG6000 solution where all seeds of wild type could not even germinate. Pierre et al. [63] conducted a field trial to test the performance of 14 transgenic wheat lines (variety Bobwhite) overexpressing the *DREB1A* gene as described by Pellegrineschi et al. [62]. Results indicated that the transgenic lines showed no unwanted pleiotropic effect as compared to control lines and the *DREB1A* gene increased the survival rate and water use efficiency. Therefore, it might be possibly assumed that high-yielding transgenic wheat lines could be achieved by adequate screening and transformation protocols.

Jiang et al. [61] investigated miRNAs in seeds of non-transgenic varieties and *GmDREB1* transgenic wheat line using deep sequencing and bioinformatic techniques. They found that 23 miRNAs were found differentially expressed in transgenic wheat seeds. The variations between transgenic and non-transgenic wheat lines were higher than which was induced by transgene.

### 6.2. WRKY2

The WRKY proteins form one of the largest families of transcription factors in plants, characterized by their WRKY domain of about 60 amino acids. WRKY acts as a repressor or activator and regulate various plant growth processes as well as abiotic and biotic stress responses [67]. Various transgenic plants, arabidopsis [68] and tobacco [69] overexpressing WRKY genes from wheat conferred abiotic stress tolerance.

Okay et al. [55] investigated the expression patterns of TaWRKY proteins in the leaf and root of drought-tolerant and susceptible bread wheat cultivars under drought. Through in-silico searches, 35 WRKY transcripts belonging to 10 *TaWKRY* genes were detected and the expression of all the quantified TaWRKY transcripts was found to be up-regulated in the roots of drought-tolerant wheat under drought. Gao et al. [70] cloned the promoter of the TaWRKY2 gene from a drought-tolerant wheat ‘Xifeng20′ into a spring wheat cultivar ‘Fielder’. The over-expression of the promoter of the *TaWRKY2* gene contributes to drought tolerance as indicated by lesser water loss and higher survival rate as compared to wild type and also of spring wheat. In addition, the transgenic *TaWRKY2* wheat had a higher yield and higher chlorophyll, proline, and sugar contents [70].

### 6.3. HDG11

*HDG11*, a transcription factor belongs to the HDZip IV TF subfamily, was found to induce drought tolerance by increasing the expression of numerous genes responsive to drought, including genes involved in calcium signaling and ABA synthesis pathway [71,72]. Transgenic wheat overexpressing the *AtHDG11* gene were studied after 30 days of water stress conditions [60]. Transgenic lines overexpressing *AtHDG11* not only improved the physiological traits but also increased the yield and exhibited lower water loss rate and stomatal density, while accumulating more proline contents. The activities of antioxidative enzymes, net photosynthesis rate, water use efficiency, and grain yield were higher in transgenic wheat than non-transgenic after drought stress.

### 6.4. TaSHN1

Cuticle protects plant from various abiotic and biotic stresses and from water loss. Cuticle biosynthesis depends on various metabolic activities which are regulated by several TFs from different families [73]. SHINE1/WAX INDUCER1 is TF in Arabidopsis belongs to the APETALA2/Ethylene Response Factor family involved in the regulation of cuticle biosynthesis. Overexpression of SHINE1 increased the accumulation of wax by activating gene expression encoding enzymes involved in wax biosynthesis [74]. Bi et al. [58] reported that the activation of the *TaSHN1* promoter conferred drought tolerance in transgenic wheat without any yield loss under controlled growth conditions. The overexpression of the *TaSHN1* gene in transgenic wheat plants enhanced drought tolerance by changing cuticle composition, effecting cuticle biosynthetic genes and decreasing the leaf stomatal density as compared to non-transgenic plants [58].

### 6.5. NAC

NAC constitutes one of the largest families of transcription factors and is characterized by a highly-conserved DNA binding domain at the N-terminus (NAC domain). Several members of the NAC family have been identified in many plant species and are known to be involved in the regulation of plant response under abiotic and biotic stresses by constitutive expression [75,76]. Many NAC transcription factors have been genetically engineered in crops to improve tolerance [77,78]. Xue et al. [79] identified four highly homologous NAC genes (*TaNAC69*) in wheat. The expression of *TaNAC69* genes was highly upregulated in the leaf and root of wheat under drought stress, however, the expression of *TaNAC69* gene in roots under normal conditions indicated the involvement of the *TaNAC69* gene in root cellular activities [79]. Later, Tang et al. [80] identified six NAC transcription factors in wheat, which are grouped into development-related, stress-related and membrane-associated transcription factors. All these NAC were induced by dehydration and other abiotic stresses. The over-expression of TaNAC69 was studied in transgenic wheat under drought-inducible barley HvDhn4s promoter [77]. The constitutive over-expression of *TaNAC69* in transgenic wheat lines induced the enhanced expression of several stress-related genes. Transgenic lines produced more root biomass under PEG-induced dehydration stress [77].

### 6.6. bZIP2

The bZIP transcription factors, characterized by a highly conserved bZIP domain, are composed of the leucine zipper and amino acids. bZIP transcription factors are involved in ABA signaling and plant response to various stresses such as drought. Its overexpression increased tolerance to drought stress [80]. Several group A bZIP transcription factors have been characterized in wheat and their role in stress response of transgenic plants has been described [56,81]. bZIP transcription factor, TabZIP2, was overexpressed in transgenic wheat lines under the drought-inducible ZmRab17 promoter. Under moderate water stress, transgenic plants were smaller with a lesser number of spikes and seeds, but seed weight was increased compared to control plants. These changes were attributed to the rearrangement of carbohydrates in plant parts under drought and wrong information about the strength of drought by a higher number of TaZIP2 transcripts in transgenic plants, indicating the possible role of TaZIP in the signaling pathway [82].

## 7. Post-Translational Modification

Post-translational modification of small ubiquitin-like modifiers (SUMOylation) is an important mechanism and regulates plant growth and development under stress conditions [9,83]. SUMOylation is reversible and SUMO-substrate linkage is cleaved by SUMO protease in the deSUMOylation process. Overexpression of OVERLY TOLERANT TO SALT-1 (OTS1) protease has increased salt tolerance in arabidopsis [84], and salt and drought tolerance of transgenic rice [85]. Le Roux et al. [86] performed the first study on the SUMOylation effect in wheat by transferring the cysteine protease AtOTS1 from *Arabidopsis thaliana* to wheat. Overexpression of AtOTS1 in bread wheat improved the drought tolerance of transgenic wheat. Transgenic wheat showed improved growth and delayed senescence under drought by increasing photosynthesis and chlorophyll content.

## 8. Protein Kinase

### 8.1. Phosphoenolpyruvate Carboxylase Kinase Related Kinases

Protein kinases regulate key aspects of cellular function, including responses to external signals [87]. Phosphoenolpyruvate carboxylase kinase-related kinases (PEPKRs) belong to the CDPK-SnRK superfamily of protein kinase [87]. Drought-tolerant wheat cultivar showed higher induction of TaPEPKR2 under drought stress, while sensitive cultivar exhibited less induction of TaPEPKR2 [88]. Phosphoenolpyruvate carboxylase kinase-related kinase gene (*TaPEPKR2*) is a highly conserved serine-threonine protein kinase gene and is reported to be induced by heat and drought. Transgenic wheat lines transformed with the TaPEPKR2 gene exhibited enhanced heat and dehydration stress tolerance and higher total root lengths in the presence of 10% PEG than wild type [89].

### 8.2. Signal Transduction Genes

Calcineurin B-like (CBLs) proteins are Ca^2+^ sensor proteins that under unfavorable conditions and drought stress interact with target proteins to transduce signals. CBLs proteins interact with CBL-interacting protein kinase (CIPKs) [90]. Cui et al. [91] reported that 21 *TaCIPK* genes were overexpressed in wheat under drought, and *TaCIPK23* was most responsive. Transgenic wheat over-expressing *TaCIPK23* genes showed a higher survival rate under drought stress with a higher accumulation of osmoprotectants, and the expression of drought and ABA-responsive genes was increased.

## 9. Nuclear Factor

Nuclear factor Y (*NF-Y*) gene family is involved in various regulatory functions for plant development and performance under stress [92]. In heaxaploid wheat, 80 NF-Y genes have been identified, but the exact number of NF-Y genes is still unknown [93].

Nelson et al. [94] reported that transgenic maize-overexpressing *ZmNF-YB2* showed improved growth under drought stress. Yadav et al. [95] isolated *NF-YB* and *NF-YC* encoding genes from drought-tolerant *Triticum aestivum* cultivar RAC875, and *TaNF-YB4* was placed in wheat cultivar Gladius under constitutive polyubiquitin maize promoter by using biolostic bombardment. The effect of overexpression of *TaNF-YB4* gene in T2 lines was investigated under mild water stress. T2 lines overexpressing *TaNF-YB4* indicated a greater number of spikes under water stress whereas other yield components were not increased. Although under well-watered conditions, a significant increase in yield was observed [95] (Table 1).

## 10. Limitations

Progress for successful development of drought-resistant wheat depends on the knowledge of functional genomics. Drought resistance is a complex trait and it is necessary to identify the function and structure of key genes in the development of wheat plants for drought tolerance and finally for higher grain yield. Plant biologists could use this knowledge to alter the structure and functions of selected genes through genetic manipulation.

Less progress has been made for the development of transgenic wheat, and a number of reasons could explain the lack of success. Wheat has a complex and large hexaploid genome, almost five times greater than the human genome which contains almost 128,000 genes [102], with 80–85% of them being repetitive sequences of DNA due to hexaploidy genome [103]. The genetic transformation efficiency of wheat is low and dependent on genotypes. Most of the studies have focused on the survival rate of plants under severe water stress which rarely has importance for wheat and other crops [104]. In some cases, the survival rate is accompanied with undesirable phenotypic features such as reduced plant size and yield [100,105,106].

The development of drought tolerance by transforming a single gene seems unsatisfactory. A single gene is easier to manipulate however, it is less suitable to confer tolerance under various field conditions. Thus, multiple genes should be considered to manipulate the built-in feedback control mechanism and to evade the overaccumulation of intermediate products. Moreover, the wheat transformation may generate completely new interactions between genes making them function differently from what would be expected. There is a need to account for negative interactions between the drought tolerance and other traits such as the photosynthetic rate in order to get a high yield. For instance, transforming homeodomain-leucine zipper I (HD-Zip I) transcription factors into wheat, the plants showed improved resistance to drought but also exhibited undesirable phenotypic characteristics such as reduced biomass and yield [105,106].

In many previous studies, molecular biologists are screening transgenic plants in small pots under severe water stress applied for few hours or days and injury or survival-related plant responses were induced under the conditions of severe stress, which will be different from the response for a long-term slowly-induced water deficit stress. However, under natural conditions, water deficit stress advances slowly in a gradual manner with the drying of soil and plants under these conditions adjusting the morphological and physiological traits accordingly, like the accumulation of osmolytes to maintain cell hydration [15]. Thus, it is important that experiments must be under conditions similar to the field. Furthermore, the intensity and duration of stress are key factors for screening which have been ignored.

Most of the studies which reported the yield increase of transgenic plants were conducted in the greenhouse-controlled conditions [96] and the response of specific transgene could be reversed under field [104]. Therefore, transgenic lines failed to sustain the benefits observed under control conditions in field trials [104]. The 14 transgenic wheat lines with DREB1A gene, selected under control conditions for high survival and water use efficiency, failed to show improved yield under water deficit in the field [63]. Only some of the researches have confirmed the improved drought resistance of transgenic wheat under field conditions [16].

Accurate phenotyping is most crucial for drought tolerance and should be selected under suboptimal field conditions in order to draw correct assumptions about the role of the discovered genes towards drought tolerance and their utilization in plant breeding. The selection of phenotypic traits for water use efficiency, osmotic adjustment, root architecture, stomatal conductance, carbohydrates remobilization, chlorophyll contents, and traits related to stay green and delayed leaf senescence will be useful for the improvement of yield under water stress [56].

Furthermore, there are some significant issues related to the cascading effect of the regulatory gene on different genes pathways, such as increased competition due to introgression of a transgene into wild, and the impact of regulatory genes on the environment and human health, which need to be properly addressed [15].

## 11. Future Directions in the Development of Drought-Tolerant Transgenic Wheat

Genetic modification is a promising approach which gives us an insight into the regulatory mechanism by a genetic change of a single or few genes. Recently, some progress has been made by transgenic approaches in identifying the key regulators of drought tolerance in wheat. Transgenic wheat plants with inserted genes such as structural and protected genes of mannitol, proline, glycine betaine, and LEA protein and regulatory genes of NAC and DREB transcription factors are being used for drought tolerance. The first drought-tolerant genetically-modified wheat was developed by the Argentine company, but needs approval for commercial release. After the approval for commercial release, Argentina will be the first country to release genetically-modified wheat engineered for drought tolerance [107].

It is of importance that the tolerance to a specific stress such as water stress be considered with the tolerance to other stresses. Wheat has a complex hexaploid allopolyploidy genome structure. Therefore, future breeding and genetic transformation efforts require the complete information of functional genomics and identification of the functional and structural role of genes involved in tolerance and determining higher grain yield. Some potential genes have been discovered which have not yet been tested in wheat and could be targeted to improve drought tolerance and yield. These potential genes are responsible for allocating sucrose during seed development, recovering from drought stress at the vegetative stage, and signaling genes involved in plant growth regulations. Research is being undergone in Mexico by the International Maize and Wheat Improvement Centre in which genetically-modified wheat inserted with these genes will be studied, and the yield will be determined under drought. Furthermore, genetic variations in wheat genotypes for sucrose allocation will be focused. Hopefully, the new developed drought-tolerant varieties will soon be developed and deployed through exchanging knowledge between research institutes and industries. Some modern and advanced molecular techniques like CRISPR/Cas9 and RNA interference are emerging as new end-products-based regulators in stress tolerance. These approaches could provide a more favorable potential base for future wheat-breeding programs by effective collaboration with traditional breeders. By collaboration and successful introgression of these genetic techniques, the outcome could be translated from laboratory tools to fields.

## Figures and Tables

**Table 1:** Improving drought tolerance of wheat through engineering gene.

Transgene	Transgenic Recipient	Source	Improved Traits	References
*TaWRKY2*	Fielder, a spring *Triticum aestivum* cultivar	Xifeng20, a drought tolerant wheat	Higher survival rate, proline, soluble sugar and chlorophyll.	[70]
calcineurin B-like protein (CBL)-interacting protein kinase *CIPK23*	Fielder, a *Triticum aestivum* cultivar	*Triticum aestivum* cultivar Xiaobaimai	Higher survival rate, increased osmolytes, induction of stomatal closure, enhanced ABA sensitivity.	[91]
aldose reductase gene *MsALR*	CY-45, a spring *Triticum aestivum* cultivar	Alfalfa	Higher detoxification activity for the aldehyde substrate; higher biomass and seed weight.	[96]
*HVA1*	Hi-Line, a spring *Triticum aestivum* cultivar	Barley	Improved biomass and water use efficiency.	[48]
*HVA1*	Hi-Line, a spring *Triticum aestivum* cultivar	Barley	Higher plant height, total biomass and grain yield.	[49]
Mannitol-1-phosphate dehydrogenase *mtlD*	Bobwhite, *Triticum aestivum* cultivar	*Escherichia coli*	Improved biomass, mannitol accumulation.	[18]
*betA* encoding choline dehydrogenase	Jinan 17, *Triticum aestivum* cultivar	*Escherichia coli*	Accumulation of glycinebetaine.	[32]
Betaine aldehyde dehydrogenase, *BADH*	*Triticum aestivum*	*Atriplex hortensis*	Higher BADH activity, show normal growth.	[35]
Betaine aldehyde dehydrogenase, *BADH*	Line (T6), from Shi4185 line	*Atriplex hortensis*	Accumulation of glycinebetaine.	[31]
Betaine aldehyde dehydrogenase, *BADH*	Line (T6), from Shi4185 line	*Atriplex hortensis*	Decreased PSII photoinhibition.	[36]
*GmDREB*	Lumai22, *Triticum aestivum* cultivar	*Glycine max cultivar Jinong27*	Improved drought tolerance with more leaves, roots and high soluble sugar contents.	[17]
Δ^1^-pyrroline-5 carboxylate synthetase, *P5CS*	CD200126, *Triticum aestivum* cultivar	*Vigna aconitifolia*	Proline biosynthesis.	[19]
Δ^1^-pyrroline-5 carboxylate synthetase, *P5cs*	*Triticum aestivum*	*Triticum aestivum*	Proline accumulation.	[28]
*DREB1A*	bread wheat	*Arabidopsis thaliana*	More branched root phenotype higher total number of heads, enhance drought tolerance.	[62]
sedoheptulose-1, 7-bisphosphatase *SBPase*	Line (T2) from cultivar Cadenza	*Brachypodium distachyon*	SBPase promoter fully drive the GUS expression.	[97]
*HaHB4*	cv. Cadenza	Sunflower	Increased yield and water use efficiency.	[16]
*AtWRKY30*	Sakha-61 genotype, *Triticum aestivum*	*Arabidopsis thaliana*	Higher biomass, photosynthesis, relative water content, prolines, soluble proteins, soluble sugars, and antioxidant enzymes activities.	[98]
*AtHDG11*	Chinese Spring, *Triticum aestivum*	*Arabidopsis thaliana*	More yield, higher proline content and photosynthesis, lower stomatal density, lower water loss rate, and increased activities of catalase and superoxide dismutase.	[60]
cold shock protein gene *SeCspA*	cultivar KN199, winter wheat	*Escherichia coli*	Higher proline, grain weight and grain yield, less reduction in chlorophyll, low MDA content.	[44]
ferritin gene, *TaFER-5B*	Jimai5265, wheat cultivar	wheat cultivar, TAM107	Improved leaf iron content and ROS, enhanced drought and temperature tolerance.	[51]
phosphoenolpyruvate carboxylase kinase-related kinase gene, *TaPEPKR2*	Liaochun10, wheat cultivar	wheat cultivar, TAM107	Enhanced drought tolerance, higher root length.	[89]
*TaSHN1*	*Triticum aestivum* cultivar Gladius	Australian drought tolerant genotype RAC875	Lower stomatal density and leaf water loss, and improved recovery after severe drought.	[58]
*TaNF-YB4*	*Triticum aestivum* cultivar Gladius	*Triticum aestivum* cultivar RAC875	More spikes.	[95]
*DREB/CBF* gene *TaRAP2.1Lmut*	*Triticum aestivum* cultivar Gladius	*Triticum aestivum* cultivar RAC785	Enhanced ability to survive frost and drought.	[99]
*OTS1,* overly tolerant to salt-1	*Triticum aestivum* Gamtoos-R	*Arabidopsis thaliana*	Delayed senescence, higher relative water content, photosynthesis and antioxidants.	[86]
*TaNAC69*	*Triticum aestivum* cultivar Bobwhite	*Triticum aestivum*	More root biomass, longer roots.	[77]
*TabZIP2*	*Triticum aestivum* cultivar Gladius	*Triticum aestivum* cultivar RAC875	Fewer spikes and seeds, increased single seed weight.	[82]
*DREB*	*Triticum aestivum* cultivar Bobwhite	*Triticum durum* L. cultivar Langdon	Improved survival, slow growth, delayed flowering, less grain yield.	[100]
*DREB*	*Triticum aestivum* cultivar 8901, 5–98, 99–92, Baofeng 104	*Arabidopsis thaliana*	Still green after 15 d withholding water, high proline contents.	[101]
*PEPC*	*Triticum aestivum* cultivar Zhoumai19	Maize	Higher proline, soluble sugar and water use efficiency.	[53]
*CspA* and *CspB*	*Triticum aestivum* cultivar KN199	*Escherichia coli*	Lower water loss rate and MDA content, higher chlorophyll, proline and yield.	[44]
